# A note on the Hendrickson–Lattman phase probability distribution and its equivalence to the generalized von Mises distribution

**DOI:** 10.1107/S1600576724000311

**Published:** 2024-02-16

**Authors:** Michael J. Barnett, Richard L. Kingston

**Affiliations:** a School of Biological Sciences, University of Auckland, Auckland, New Zealand; Uppsala University, Sweden; The European Extreme Light Infrastucture, Czechia

**Keywords:** crystallographic phase determination, probability density functions, circular statistics, computational crystallography

## Abstract

Hendrickson & Lattman [*Acta Cryst.* (1970), B**26**, 136–143] introduced a method for representing crystallographic phase probabilities defined on the unit circle. Their approach could model the bimodal phase probability distributions that can result from experimental phase determination procedures. It also provided simple and highly effective means to combine independent sources of phase information. The present work discusses the equivalence of the Hendrickson–Lattman distribution and the generalized von Mises distribution of order two, which has been studied in the statistical literature. Recognizing this connection allows the Hendrickson–Lattman distribution to be expressed in an alternative form which is easier to interpret, as it involves the location and concentration parameters of the component von Mises distributions. It also allows clarification of the conditions for bimodality and access to a simplified analytical method for evaluating the trigonometric moments of the distribution, the first of which is required for computing the best Fourier synthesis in the presence of phase, but not amplitude, uncertainty.

## Abstract 2

The equivalence between the Hendrickson–Lattman phase probability distribution and the generalized von Mises distribution of order two is documented using both formulae and figures.

## 1. Introduction

To enable determination of protein structures using X-ray crystallography, a variety of methods for experimental phase determination were developed and refined over several decades [see Hendrickson (2023 ▸) for a review]. All of these methods involved the systematic perturbation of Bragg diffraction from the crystal, by manipulating either the chemical composition of the crystal, the physical properties of the irradiating X-rays or both. A well understood feature of some approaches to experimental phase determination, including single isomorphous replacement and single wavelength anomalous dispersion, is that a twofold geometric ambiguity in the phase results, even in the absence of error (Matthews, 1970 ▸; Vijayan, 1980 ▸; Dauter *et al.*, 2002 ▸; McCoy & Read, 2010 ▸; Hendrickson, 2014 ▸). Hence the practical application of these phase determination procedures naturally generates bimodal phase probability distributions. This complexity must be captured in any mathematical function used to represent these probabilities. In addition, resolving the crystallographic phase problem for biological molecules experimentally often requires the combination of phase information from independent experiments.

The probability density function introduced by Hendrickson & Lattman (1970 ▸) addressed these issues. It has the form

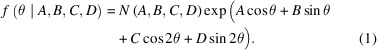

Here (*A*, *B*, *C*, *D*) are the four coefficients of the distribution, which encode the phase information, and *N* is a normalization constant. Depending on the values of the coefficients, (1) may be either unimodal or bimodal. Most conveniently, when (1) is used to represent phase probabilities, independent sources of phase information can be combined through simple addition of the coefficients (*A*, *B*, *C*, *D*) because of the exponential form of the distribution.

Hence, (1), being both sufficiently flexible and numerically very convenient, became widely used to represent phase probability distributions in protein crystallography. We note that the Hendrickson–Lattman distribution is useful for modeling the phase probability distributions of acentric data, where the phase can take any value in the range 0–2π. For centric data, where there are only two phase possibilities, always separated by π, a discrete circular probability mass function provides the most straightforward descriptor.

Although the treatment of error in experimental phase determination has become increasingly sophisticated and is now generally based on the principle of maximum likelihood, with joint consideration of uncertainty in both amplitudes and phases (Read, 2003 ▸; Bricogne *et al.*, 2003 ▸; McCoy & Read, 2010 ▸), the Hendrickson–Lattman distribution is still used to represent phase probability distributions in modern crystallographic software. Hence some clarification of its basic characteristics seems worthwhile.

Hendrickson & Lattman (1970 ▸) briefly noted the similarities between their probability density distribution and the von Mises distribution, and the connection has been remarked on subsequently (Murshudov *et al.*, 2011 ▸). However, to the best of our knowledge, these observations have not been systematically developed. Fully documenting the relation between the Hendrickson–Lattman and von Mises distributions and placing the procedures used by Hendrickson & Lattman (1970 ▸) within the framework of circular statistics is the purpose of this short review.

## 2. The von Mises distribution

**Figure 1:**
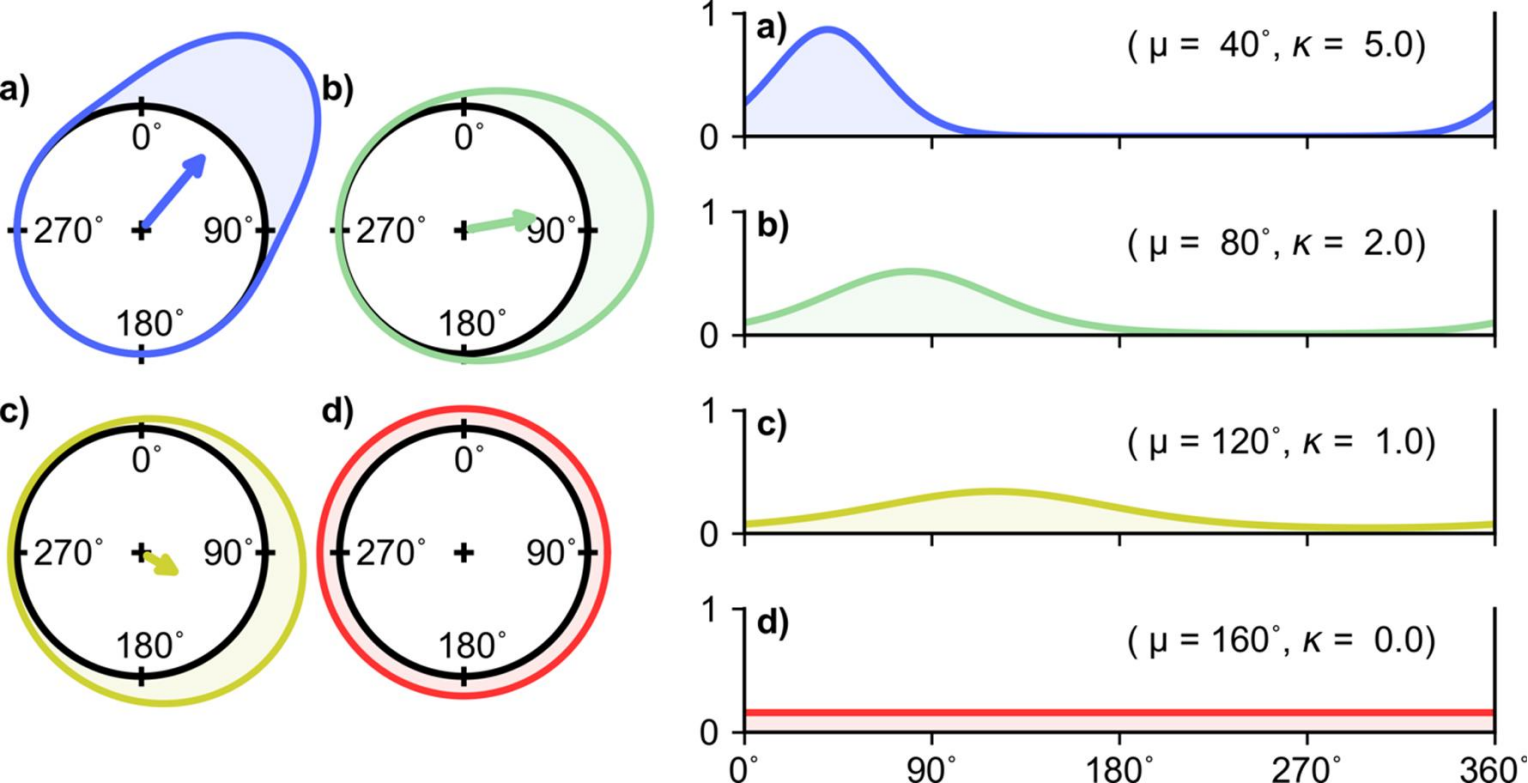
The von Mises distribution. (*a*)–(*d*) Four different instantiations of the von Mises probability density function represented in (left) circular form and (right) linear form, where the functions have been unwrapped onto the line. In the circular representation, the radial distance from the unit circle at each angle indicates the probability density (solid shaded), and the vectors internal to the unit circle display the first trigonometric moment of the probability density distribution, calculated according to (13), which identifies its center of mass. When κ = 0, the von Mises distribution reduces to the uniform circular distribution. In this case, the center of mass of the distribution corresponds to the center of the unit circle, and the first trigonometric moment is not fully defined.

The von Mises probability density function is central to circular statistics, being the circular analog of the Gaussian probability density function on a line, and its properties are consequently very well documented (Batschelet, 1981 ▸; Fisher, 1993 ▸; Mardia & Jupp, 1999 ▸; Jammalamadaka & Sengupta, 2001 ▸). Like the Gaussian, the von Mises distribution is a mirror symmetric mono-modal distribution, defined by two parameters (Fig. 1 ▸). μ is a location parameter. The function takes on its maximum value at μ, which is both the modal and mean value of the distribution. κ is a concentration parameter, named because as κ increases the distribution becomes more concentrated around μ. The von Mises probability density function is given by

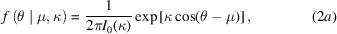

where μ ∈ [0, 2π), κ ≥ 0, and *I*_0_ is the modified Bessel function of the first kind and order 0.

**Figure 2:**
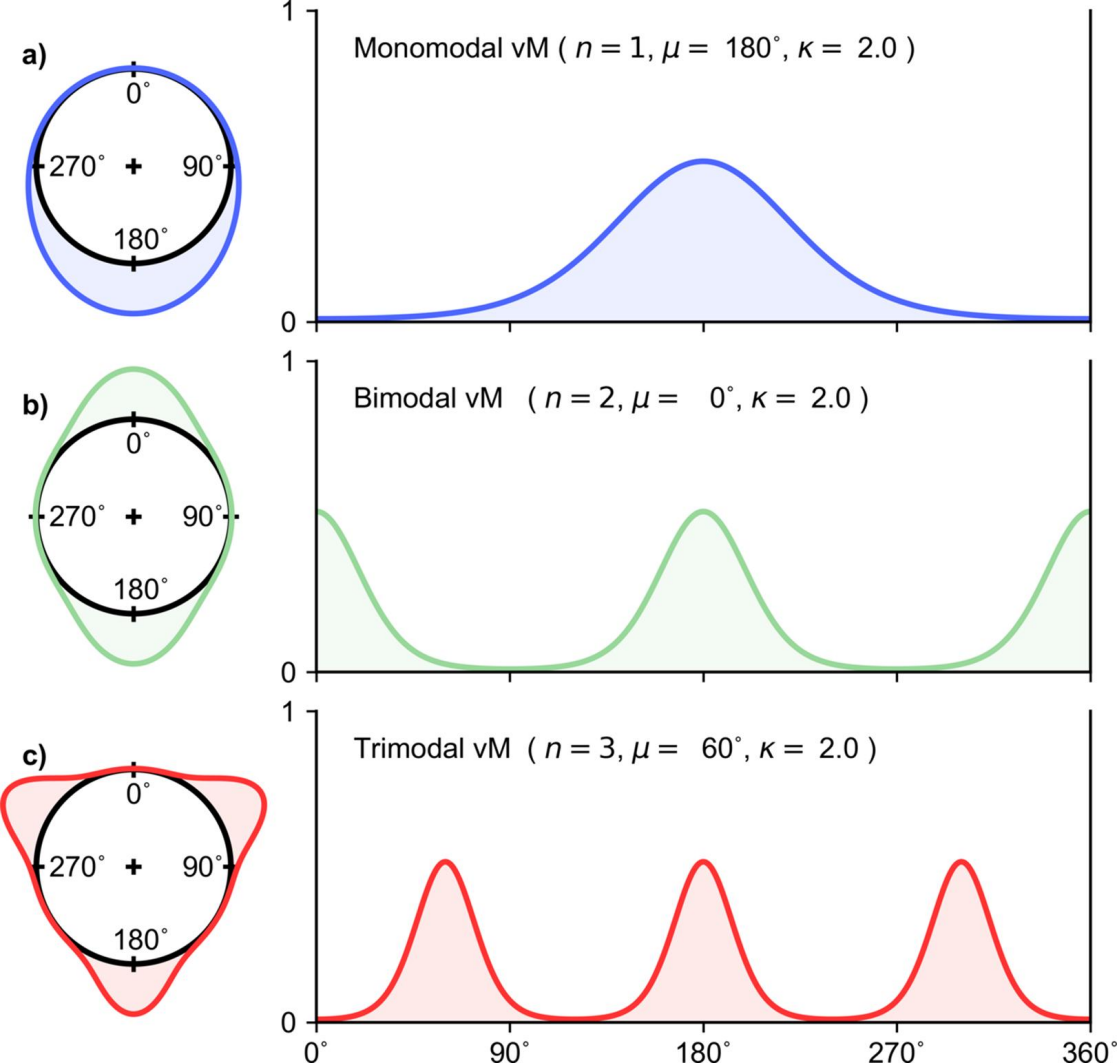
Multimodal von Mises distribution. A single instantiation of the (*a*) monomodal (*n* = 1), (*b*) bimodal (*n* = 2) and (*c*) trimodal (*n* = 3) von Mises probability density functions are represented in (left) circular form and (right) linear form, as in Fig. 1 ▸. When *n* = 1, the ordinary von Mises distribution results (see Fig. 1 ▸).

A simple extension of the von Mises distribution allows for multimodality, subject to symmetry restrictions (Mardia & Spurr, 1973 ▸; Batschelet, 1981 ▸). The multimodal von Mises probability density function is given by

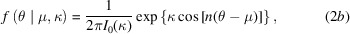

where μ ∈ [0, 2π/*n*), κ ≥ 0 and *n* is a positive integer that specifies the number of modes. The modes of this highly symmetric distribution are separated by 2π/*n*, as depicted in Fig. 2 ▸ for the monomodal (*n* = 1), bimodal (*n* = 2) and trimodal (*n* = 3) cases.

## 3. The generalized von Mises distribution and its equivalence with the Hendrickson–Lattman distribution

**Figure 3:**
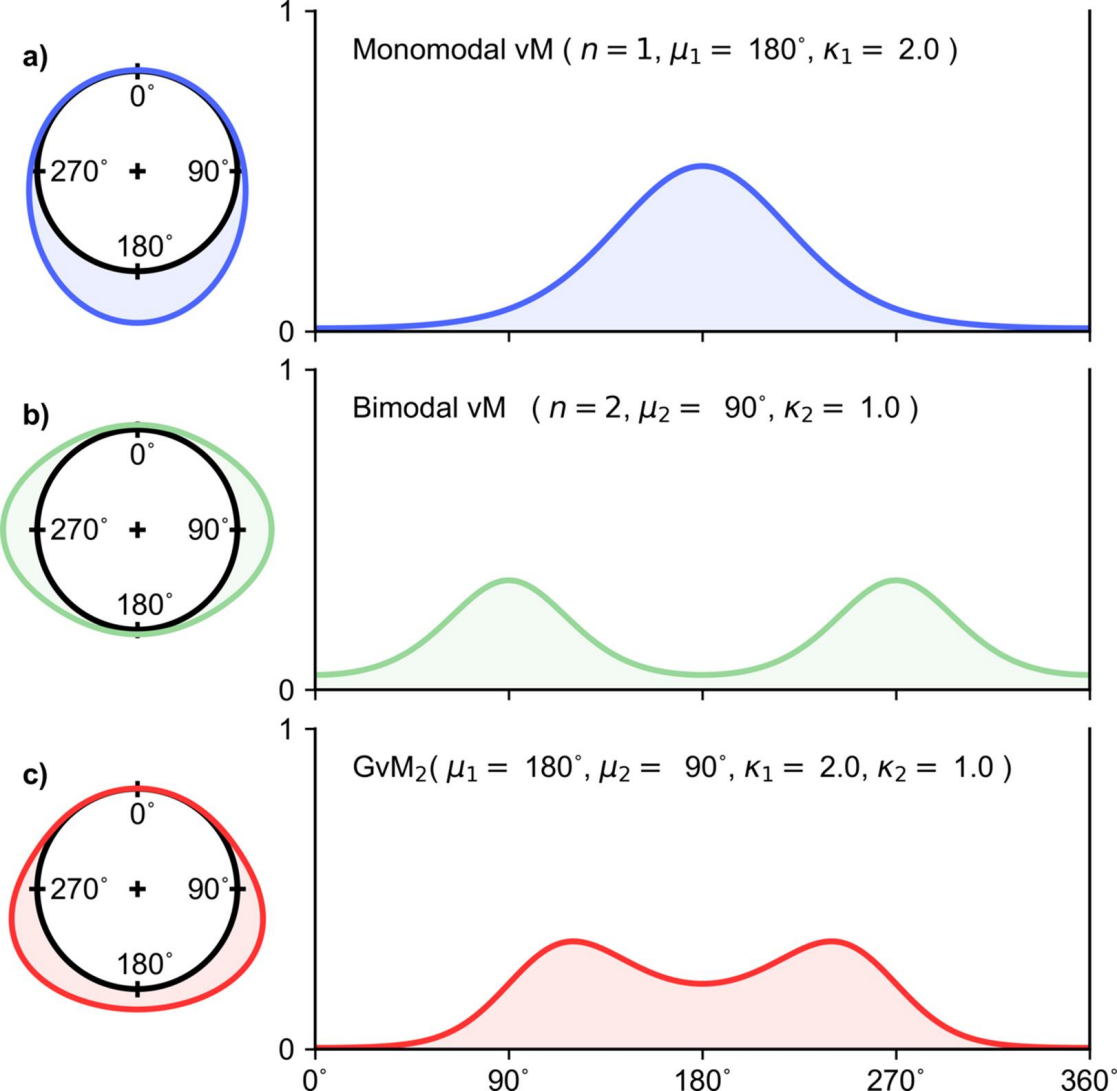
Construction of the generalized von Mises distribution of order 2, from the monomodal and bimodal von Mises distributions. (*a*) Instantiation of the monomodal von Mises distribution with the parameters (μ_1_, κ_1_) as indicated. (*b*) Instantiation of the bimodal von Mises distribution with the parameters (μ_2_, κ_2_) as indicated. (*c*) Generalized von Mises distribution with the parameters (μ_1_, μ_2_, κ_1_, κ_2_). This is the product of the unimodal and bimodal distributions shown in (*a*) and (*b*), normalized by the constant [*I*_0_(κ_1_)*I*_0_(κ_2_)]/*G*_0_(μ_1_, μ_2_, κ_1_, κ_2_). The distributions are represented in (left) circular form and (right) linear form as in Fig. 1 ▸.

The ordinary von Mises distribution [equation (2*a*), Fig. 1 ▸] is both unimodal and mirror symmetric, whereas its multimodal extension [equation (2*b*), Fig. 2 ▸] has both mirror and rotational symmetry. This limits applications. An important generalization of the von Mises distribution (Gatto & Jammalamadaka, 2007 ▸), which allows for both bimodality and asymmetry, is given by

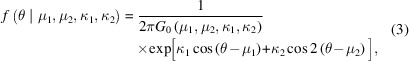

where μ_1_ ∈ [0, 2π) and μ_2_ ∈ [0, π) are location parameters, and κ_1_ ≥ 0 and κ_2_ ≥ 0 are concentration parameters. The distribution (3) can be considered to arise from the multiplication of a unimodal and a bimodal von Mises distribution (Fig. 3 ▸), and is hence termed the generalized von Mises distribution of order 2 (the GvM_2_ distribution). Incorporating multimodal von Mises distributions of higher order into the product gives rise to an infinite series of probability distributions [see Gatto & Jammalamadaka (2007 ▸) and Gatto (2009 ▸) for context and commentary]; however, GvM distributions with order greater than two have found limited practical applications. The GvM_2_ distribution (3) appears to have been first proposed by Maksimov (1967 ▸) and its properties are now well studied (Yfantis & Borgman, 1982 ▸; Gatto & Jammalamadaka, 2007 ▸; Gatto, 2008 ▸, 2009 ▸; Salvador & Gatto, 2022*a*,*b* ▸).

The normalizing constant *G*_0_ appearing in (3) ensures that the distribution is a probability density function, and is obtained by definite integration of the function over the unit circle. This integral cannot be evaluated in closed form, but can be written in terms of an infinite series expansion (Yfantis & Borgman, 1982 ▸; Gatto & Jammalamadaka, 2007 ▸) as

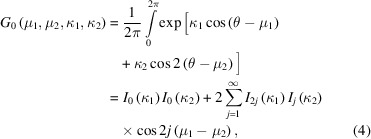

where *I_n_* are the modified Bessel functions of the first kind and integer order *n*. The derivation of this result relies on the Jacobi–Anger expansion (Olver *et al.*, 2010 ▸):

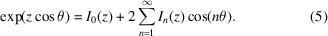

As the modified Bessel functions decrease rapidly to zero with increasing order (Oldham *et al.*, 2009 ▸), accurate evaluation of the normalizing constant *G*_0_ using (4) can be achieved with only the first few summands of the infinite series.

The GvM_2_ distribution (3) can be symmetric or asymmetric, unimodal or bimodal, depending on its parameters (μ_1_, μ_2_, κ_1_, κ_2_) [see the literature (Yfantis & Borgman, 1982 ▸; Gatto & Jammalamadaka, 2007 ▸; Kato & Jones, 2010 ▸; Salvador & Gatto, 2022*a* ▸) for demonstration and discussion]. If κ_1_ = 0, the GvM_2_ distribution [equation (3)] reduces to a bimodal von Mises distribution [equation (2*b*) with *n* = 2], whereas if κ_2_ = 0, it reduces to a monomodal von Mises distribution [equation (2*a*); equation 2(*b*) with *n* = 1]. If κ_1_ = 0 and κ_2_ = 0, the GvM_2_ distribution reduces to the uniform circular distribution (Yfantis & Borgman, 1982 ▸). The general conditions for bimodality of the GvM_2_ distribution are elaborated below.

As written, the connections between the GvM_2_ (3) and the Hendrickson–Lattman distribution (1) are not immediately seen. However the GvM_2_ distribution can be reparameterized (Gatto & Jammalamadaka, 2007 ▸) as follows.

If

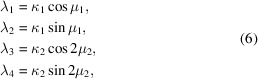

then the GvM_2_ probability density function can be expressed as

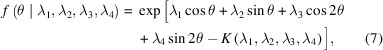

where the constant *K*(λ_1_, λ_2_, λ_3_, λ_4_) is an appropriate transformation of the normalizing factor appearing in the denominator of (3),

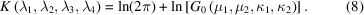

Practically, the normalizing constant *K*(λ_1_, λ_2_, λ_3_, λ_4_) [equation (8)] can be evaluated using (4). Equations (6) have the form of a polar-to-Cartesian coordinate transformation. Parameters (μ_1_, μ_2_, κ_1_, κ_2_) can therefore be recovered from parameters (λ_1_, λ_2_, λ_3_, λ_4_) using

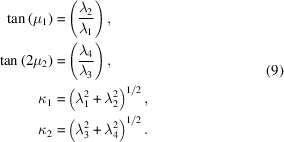

**Figure 4:**
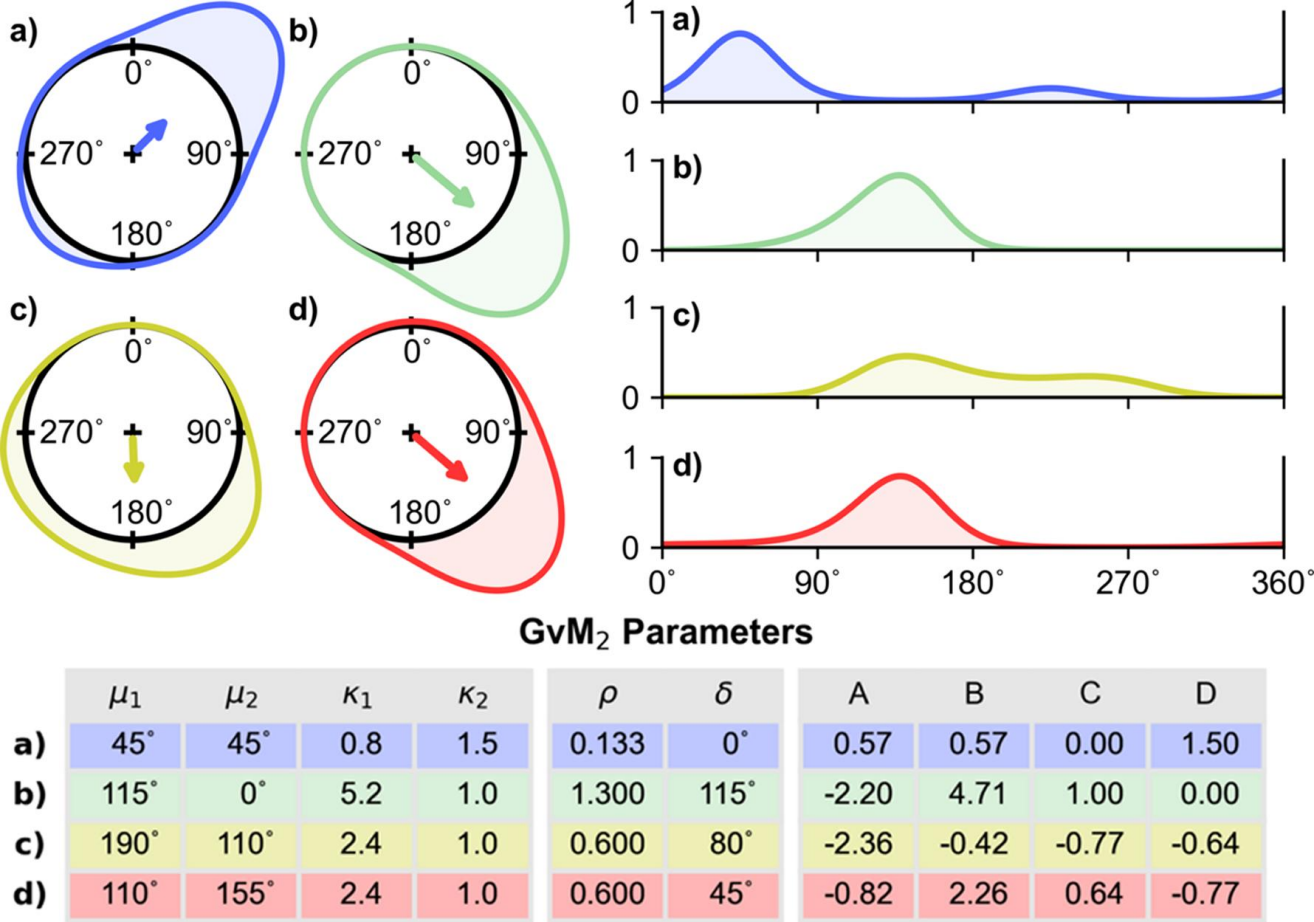
Equivalent parameterizations of the generalized von Mises distribution of order 2. (*a*)–(*d*) Four different instantiations of the GvM_2_ distribution represented in (left) circular and (right) linear form, as in Fig. 1 ▸. Each distribution can be specified using either the expanded expression (1) and the parameters (*A*, *B*, *C*, *D*) or the order factorized expression (3) and the parameters (μ_1_, μ_2_, κ_1_, κ_2_). The parameters are given in the table at the bottom of the figure. The derived parameters ρ = κ_1_/4κ_2_ and δ = μ_1_ − μ_2_ mod(π) are useful for diagnosing the bimodality of the distribution (Salvador & Gatto, 2022*a* ▸). The vectors internal to the unit circle display the first trigonometric moment of each GvM_2_ distribution, calculated according to (18).

The reparameterized version of the GvM_2_ distribution (7) is clearly equivalent to the Hendrickson–Lattman probability distribution (1), with *A* = λ_1_, *B* = λ_2_, *C* = λ_3_, *D* = λ_4_ and *N* = exp(−*K*) = 1/(2π*G*_0_). The equivalence has been noted previously (Murshudov *et al.*, 2011 ▸). The GvM_2_/Hendrickson–Lattman distributions belong to the exponential family of probability distributions, with (7) being the canonical representation of that family. The relationships between the two parameterizations of the Hendrickson–Lattman/GvM_2_ distribution are illustrated in Fig. 4 ▸. Some aspects of the distribution are easier to recognize when it is written in the order factorized form (3) rather than the expanded form of (1) or (7). For example, when μ_1_ approaches μ_2_, the GvM_2_ probability density function (3) approaches mirror symmetric, and is either unimodal or bimodal with peaks at antipodal positions [Fig. 4 ▸(*a*)] (Gatto & Jammalamadaka, 2007 ▸; Salvador & Gatto, 2022*b* ▸). When both concentration parameters (κ_1_, κ_2_) become small, the distribution approaches uniform circular.

The order factorized form of the GvM_2_ distribution (3) also allows analysis of the conditions for bimodality of the distribution, which are of particular interest in crystallography. These conditions are most readily expressed in terms of two derived quantities: the scaled ratio of the two concentration parameters, ρ = κ_1_/4κ_2_; and the difference between the location parameters, δ = μ_1_ − μ_2_ mod(π). When ρ ≤ 1/2, the GvM_2_ distribution is always bimodal [see *e.g.* Figs. 3 ▸(*c*) and 4 ▸(*a*)]. When ρ ≥ 1, the GvM_2_ distribution is always unimodal [see *e.g.* Fig. 4 ▸(*b*)]. When 1/2 < ρ < 1, the GvM_2_ distribution may be either unimodal or bimodal ,dependent on the value of δ [see *e.g.* Figs. 4 ▸(*c*) and 4 ▸(*d*)], the detail being somewhat complex as it involves the roots of a quartic equation. Full details are given by Salvador & Gatto (2022*a* ▸).

Hendrickson & Lattman (1970 ▸) actually used a functionally equivalent reparameterization of the probability distribution in their paper. To facilitate analytical integration of the distribution, and calculation of its normalizing constant *N*, they perform a change of variables, almost identical to (9), which effectively switches from the expanded form of the distribution (1) or (7) to the order factorized form (3). Allowing for the variations in definitions and notation, the result obtained for the normalization constant [equation (21*a*) of Hendrickson & Lattman (1970 ▸)] is the same as (4), up to a factor of 2π. Other integrations were performed that enable calculation of the best Fourier synthesis. Before considering these results, we first reframe the crystallographic problem being treated using the terminology of directional statistics.

## 4. The first trigonometric moment of a circular probability distribution and the best Fourier synthesis

As with probability distributions defined on the line, probability distributions defined on the circle can be characterized by a series of moments, which are obtained by integration of products of the distribution. However, these moments must be defined differently because of the circular periodicity. The trigonometric moments used to characterize circular distributions are named for the trigonometric functions that appear inside the integral. Unlike the regular moments, the trigonometric moments are complex-valued quantities. Though trigonometric moments of arbitrary order can be defined, we consider here only the first trigonometric moment which is defined as (Fisher, 1993 ▸; Mardia & Jupp, 1999 ▸; Jammalamadaka & Sengupta, 2001 ▸)

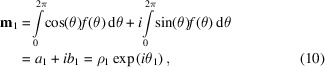

where *f*(θ) is the probability density function.

The quantities

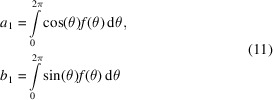

are the components of the first trigonometric moment expressed in Cartesian form.

The quantities

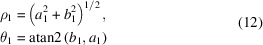

are the components of the first trigonometric moment expressed in polar form.

In the field of circular statistics, the modulus (ρ_1_) of the first trigonometric moment is termed the mean length (sometimes the mean resultant length), while the argument (θ_1_) is termed the mean direction. For the ordinary von Mises distribution (2*a*), the mean length and mean direction are given by (Fisher, 1993 ▸; Mardia & Jupp, 1999 ▸; Jammalamadaka & Sengupta, 2001 ▸)

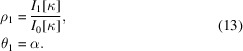

The first trigonometric moments for particular instantiations of the ordinary von Mises distribution are displayed in Fig. 1 ▸. The first trigonometric moment identifies the center of mass of a circular probability density function. The mean length, which can vary between 0 and 1, provides a useful measure of the dispersion of a unimodal distribution, such as the von Mises, though the interpretation is less straightforward for a potentially multimodal distribution such as the generalized von Mises.

Irrespective of the form of a circular probability distribution, the first trigonometric moment is of particular importance in crystallography. This is because, ignoring errors in the Fourier amplitudes, and given probability density functions for the phases, the best Fourier synthesis (in a least-squares sense) is obtained using the product of the first trigonometric moment and the measured Fourier amplitudes as coefficients. Therefore, the required coefficients are

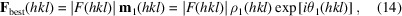

where **F**_best_(*hkl*) represents the complex Fourier coefficients and |*F*(*hkl*)| represents the measured Fourier amplitudes. Hence the best Fourier synthesis is computed using the mean direction as the phase, while weighting the Fourier amplitudes by the mean length. This is the essential result given in the foundational paper by Blow & Crick (1959 ▸) [see the literature (Matthews, 1970 ▸; Vijayan, 1980 ▸; McCoy & Read, 2010 ▸) for discussion]. In crystallographic applications, the mean length has historically been termed the ‘figure of merit’, and the mean direction the ‘best’ or ‘centroid’ phase (Matthews, 1970 ▸; Vijayan, 1980 ▸).

## 5. The first trigonometric moment of the GvM_2_ distribution

We now consider the analytical evaluation of the first trigonometric moment of the GvM_2_ distribution, which involves the integrals in (11). For the GvM_2_ distribution, no closed form solution for these integrals exists. However, as for the normalizing constant of the distribution [equation (4)], solutions can again be obtained that involve rapidly converging series expansions. For clarity, we restate the results obtained by Hendrickson & Lattman (1970 ▸), using the standard notation for the GvM_2_ distribution (3). The procedure described by Hendrickson & Lattman (1970 ▸), when applied to evaluate the integrals

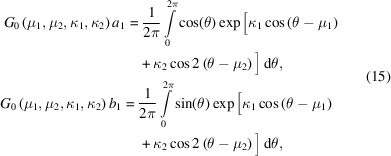

yields

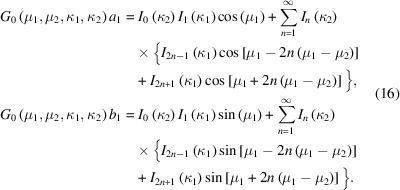

The proof again rests on the repeated use of the Jacobi–Anger expansion (5) and standard trigonometric identities. By making substitutions that reflect the variant re-parameterization of the probability density function used by Hendrickson & Lattman (1970 ▸),

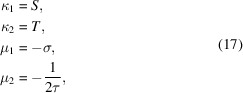

then expressions (16) are seen to be equivalent to the equations appearing at the bottom of page 141 of the article by Hendrickson & Lattman (1970 ▸), up to a factor of 2π (noting the presence of a typographical error resulting in an erroneous change of sign when specifying the Bessel functions). The result (16) can also be obtained from the expressions for the trigonometric moments of arbitrary order, reported by Yfantis & Borgman (1982 ▸), who used an identical method of derivation.

Without loss of generality, we now consider the case where μ_1_ = 0. For any GvM_2_ distribution this can be achieved by an angular coordinate transformation. When setting μ_1_ = 0, expressions (16) for the components of the first trigonometric moment simplify to

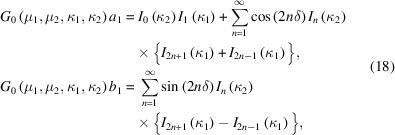

where δ = μ_1_ − μ_2_ is the difference between the location parameters of the distribution. This is a computationally more convenient way to analytically evaluate the integrals, and is also the result given by Gatto (2009 ▸), made specific for the first trigonometric moment.

The first trigonometric moments for particular instantiations of the GvM_2_ distribution, evaluated using (18), are displayed in Fig. 4 ▸.

## 6. Conclusions

Directional data are ubiquitous in the physical and biological sciences, so it is probably unsurprising that the circular probability distribution developed by Hendrickson & Lattman (1970 ▸) was independently discovered and characterized by others. The exponential form of the Hendrickson–Lattman probability distribution confers many desirable properties. However, the Hendrickson–Lattman coefficients *A*, *B*, *C* and *D* lack straightforward meaning. Recognizing the equivalence of the Hendrickson–Lattman and GvM_2_ distributions allows reparameterization of the distribution to a more intuitive form that reflects the relationship with the von Mises distribution. It also allows a fuller appreciation of the general mathematical and statistical properties of the distribution, including the conditions for bimodality, and access to analytical procedures for computing all its trigonometric moments. There may be applications in crystallography where the inferential properties of the Hendrickson–Lattman/GvM_2_ distribution become important (*i.e.* when the parameters of the distribution need to be inferred, on the basis of computational procedures that effectively sample phase probabilities), and these have been studied in the statistical literature.
